# Transosseous Cannula Suture Suspensionplasty for Thumb Basal Joint Arthritis: A Novel Technique

**DOI:** 10.1097/BTH.0000000000000426

**Published:** 2023-01-19

**Authors:** Mattia Carozzo, Giorgio Pajardi, Morena A. Basso, Dario Cirillo, Giovanni Balato, Francesco Smeraglia

**Affiliations:** * IRCCS Multimedica, Milan; † Department of Orthopaedic Surgery, “Federico II” University, Naples, Italy

**Keywords:** arthritis, thumb basal joint, trapeziometacarpal

## Abstract

The suture button (SB) suspension technique has become popular in the treatment of thumb basal joint arthritis, as it works as an internal mean for metacarpal stabilization, demonstrating good results with improvement in function and strength. The aim of our study is to describe a new transosseous suture suspensionplasty technique using a simple Ethibond #2 suture as a substitute for the suture button and to report the postoperative clinical outcomes. In this study, we included a total of 14 patients with 2 years follow-up. We evaluated patients with the use of the Disabilities of the Arm, Shoulder and Hand questionnaire, the Visual Analog Scale, the Kapandji test, and the key pinch strength. Patients treated with transosseous suture suspensionplasty demonstrated clinical improvement at an average follow-up of 24 months. No complications were noted immediately after the procedure or during the 2-year follow-up period.

Trapeziometacarpal (TMC) joint osteoarthritis (OA) is a common degenerative condition of the hands.^1^ When nonoperative treatments fail, multiple surgical options are available; however, to date, none have emerged as superior, although trapeziectomy has shown a lower complication rate and shorter operative time.^2^ After partial or complete trapeziectomy, the first metacarpal tends to subside into the newly created space.^3^ Different types of metacarpal stabilization procedures have been used to obviate the risk of metacarpal subsidence, such as ligament reconstruction with or without tendon interposition (LRTI),^4,5^ hematoma and distraction arthroplasty,^6^ suspensionplasty,^7^ and total or hemi-implant arthroplasty.^8–10^ These techniques have their own potential complications, such as stiffness, long recovery time, tendon adhesion or rupture, and pin-related complications.^2,4–9^ The suture button (SB) suspension technique was subsequently proposed to provide an internal means of metacarpal stabilization. The SB suspensionplasty is a version of hematoma distraction arthroplasty utilizing an internal device to suspend and stabilize the metacarpal.^11^ This obviates the need of K-wire stabilization, ligament reconstruction, and tendon interposition while allowing for earlier mobilization.^12^ Recently, this technique has become popular, demonstrating good results with improvement in function and strength.^11–14^ A concern of performing the SB technique is cost-effectiveness when compared with traditional techniques,^12,15^ especially when compared with similar techniques using pins or tendon interpositions. The aim of our study was to describe a new technique using a simple Ethibond #2 as a substitute for the SB.

## ANATOMY

The thumb carpometacarpal joint is a saddle joint. The metacarpal base has a volo-dorsal concavity and a latero-medial convexity. The distal surface of the trapezium has a concave latero-medial arch and a convex arch dorso-volar. This sophisticated anatomy allows the complete arcs of motion in flexion-extension and abduction-adduction. The prone-supination of the first ray occurs through a rotation and translation movement of this joint. The dorsal ligament complex is composed of 3 ligaments (dorso-radial, dorsal, and posterior oblique); this complex represents the primary restraint to dorsal dislocation and is thicker than the anterior capsuloligamentous complex. The 2 volar ligaments (anterior oblique and ulnar collateral) are tensioned only when the thumb is placed in full extension. Movement and dynamic stabilization of the trapeziometacarpal joint depend on the activity of the intrinsic and extrinsic musculature.

Proprioception of the trapeziometacarpal joint is provided by terminal branches of the median nerve (palmar and thenar cutaneous), superficial branch of the radial nerve, and lateral cutaneous nerve of the forearm.

## INDICATIONS/CONTRAINDICATIONS

The inclusion criteria were as follows: failure of conservative treatment after at least 6 months, pain at the TMC joint, and radiologic stages II to III and IV according to Eaton-Littler. There were no contraindications to surgery. Local ethics committee approval was not required for this observational study. No external funding was received for the study. Informed consent was obtained from all patients included in the study. The procedures were performed in accordance with the Declaration of Helsinki, as revised in 2013. Patients were assessed preoperatively (T0) and postoperatively at 3 months, 6 months, 1 year, and 2 years. Patients completed the Disabilities of the Arm, Shoulder, and Hand questionnaire (0 points, no disability; 100 points, complete disability) to assess the function of the upper limb. The pain was assessed using a 10-cm visual analog scale. The scale was graded from 0 to 10 cm, with 0 cm indicating no pain and 10 cm indicating maximum pain. Thumb motion was assessed using the Kapandji test, with 1 indicating incapacity of opposition and 10 indicating complete opposition, and radial and palmar abduction were measured with a goniometer. Key pinch strength was measured using a Jamar pinch dynamometer (FEI, Irvington, NY) on both the operated and non-operated hands. Clinical assessments were performed by a single senior orthopedic resident with adequate training in hand surgery. Complications and reoperations were also recorded.

## TECHNIQUE

The procedure was performed with patients awake or under regional anesthesia. The patient was placed in the supine position, with the forearm and hand supine. An upper arm tourniquet was inflated to 250 mm Hg.

**FIGURE 1:**
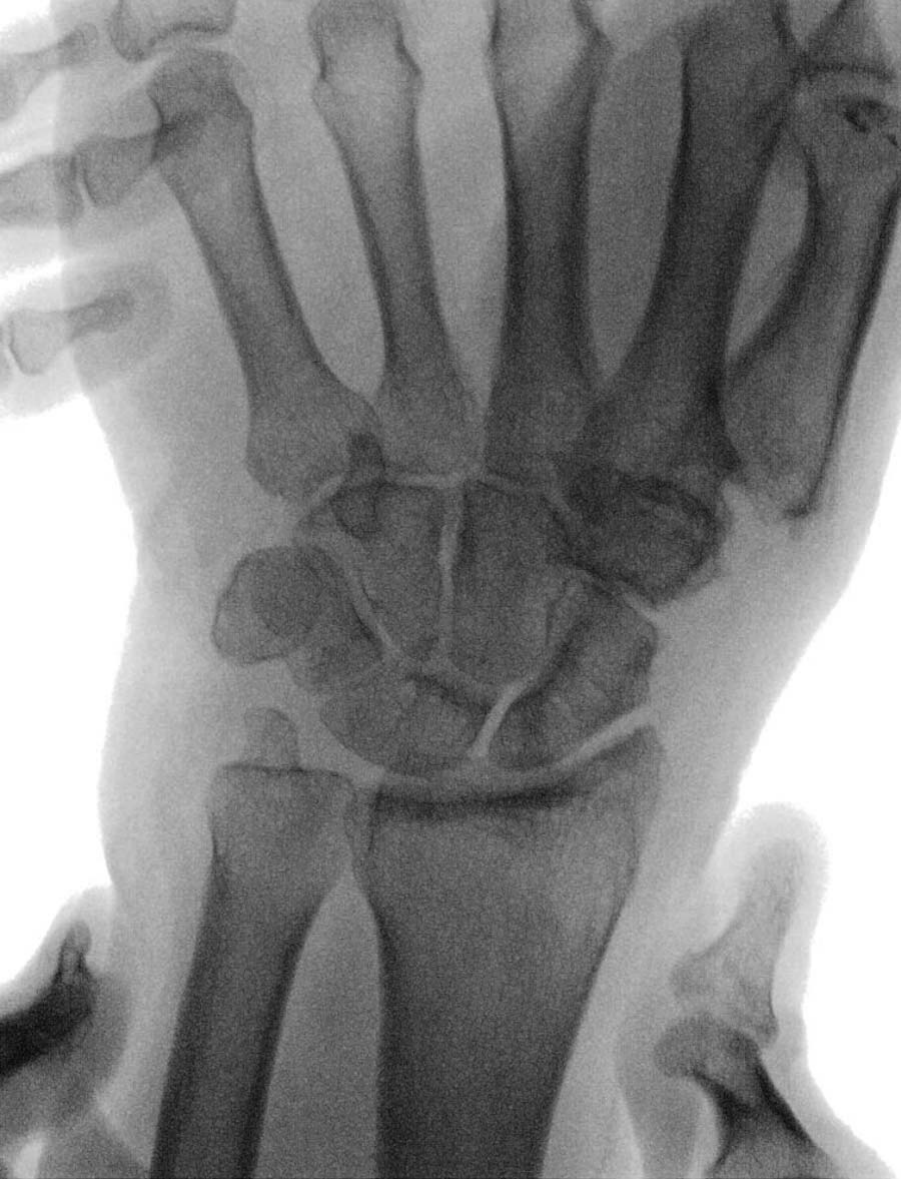
Preoperative x-ray.

**FIGURE 2:**
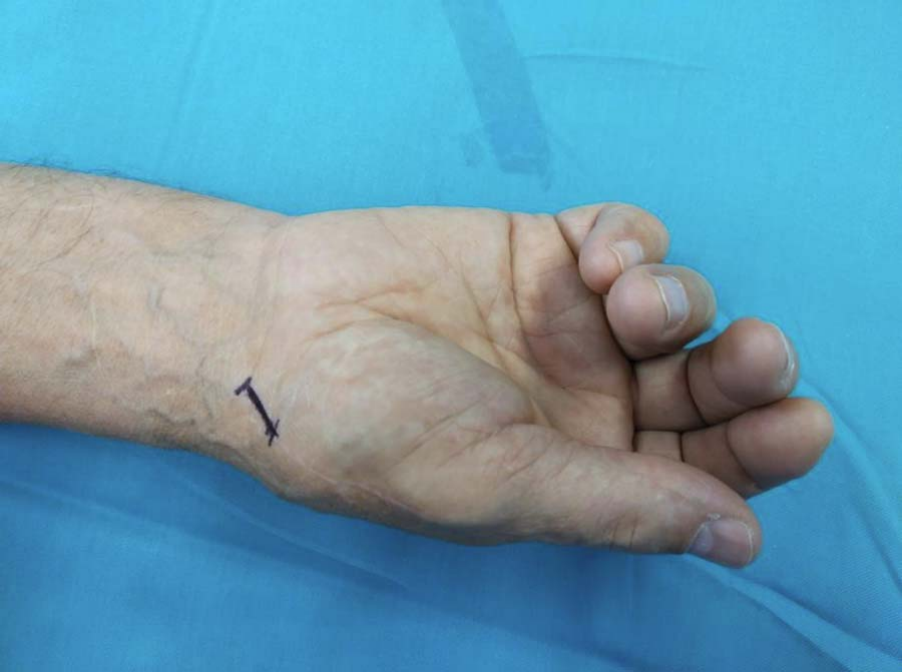
A volar skin incision starting proximally from the tubercle of the scaphoid and trapezium up to the base of the first metacarpal bone distally.

Step 1: A volar skin incision of ~2 cm was made starting proximally from the tubercle of the scaphoid and trapezium up to the base of the first metacarpal bone distally (Figs. 1, 2)

Step 2: Dissection was extended down through the subcutaneous tissues. The superficial branch of the radial nerve, which is located below the radial flap for surgical access, was identified and protected throughout the procedure.

**FIGURE 3:**
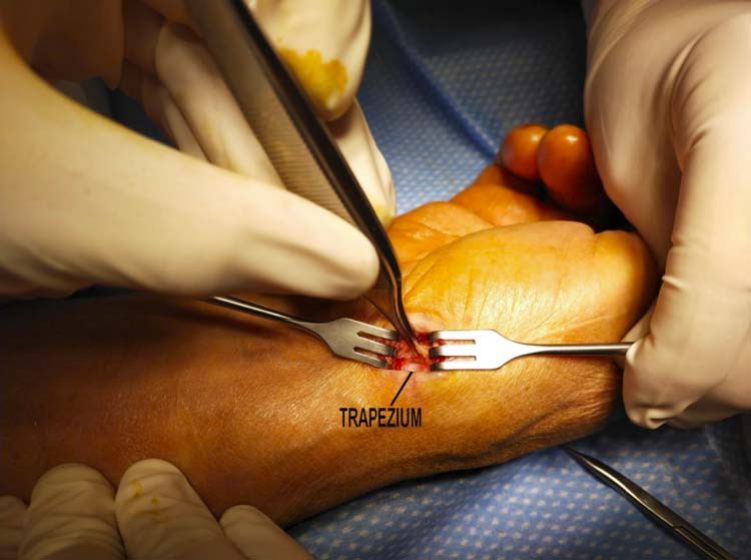
Exposition of the trapezium after the thenar muscle stripping and the basal joint capsule longitudinal incision.

Step 3: The thenar muscles were stripped from the volar aspect of the basal joint capsule, exposing the capsule, and a longitudinal incision was made in the basal joint capsule, and the trapezium was exposed (Fig. 3).

**FIGURE 4:**
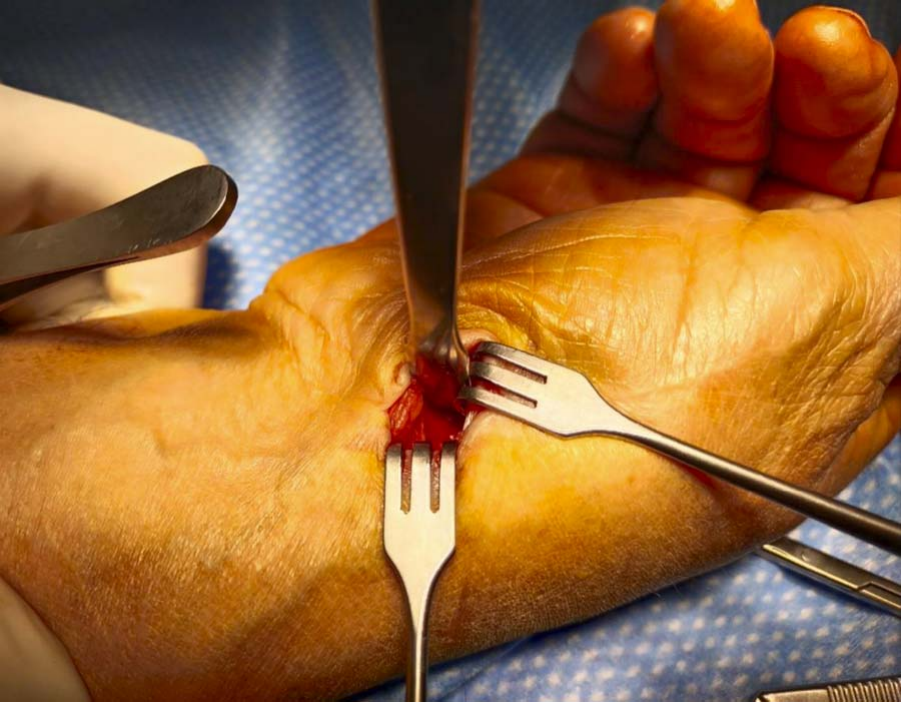
Protection of the flexor carpi radialis by inserting an Hohmann lever under the thenar muscle on the volar-ulnar corner of the trapezium.

**FIGURE 5:**
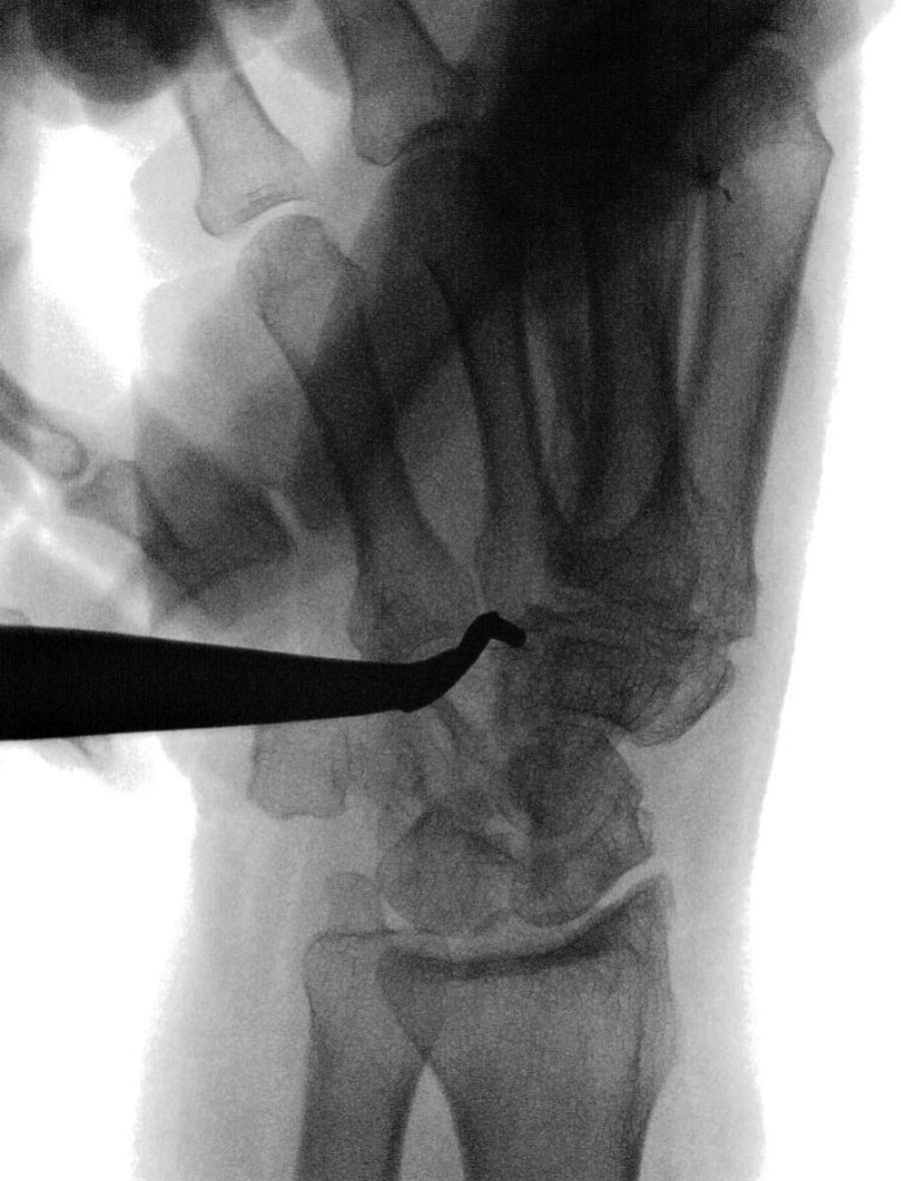
Intraoperative fluoroscopic view of the Homan lever position.

Step 4: Insert a Hohmann lever under the thenar muscle on the volar-ulnar corner of the trapezium, preverving the flexor carpi radialis (Figs. 4, 5)

**FIGURE 6:**
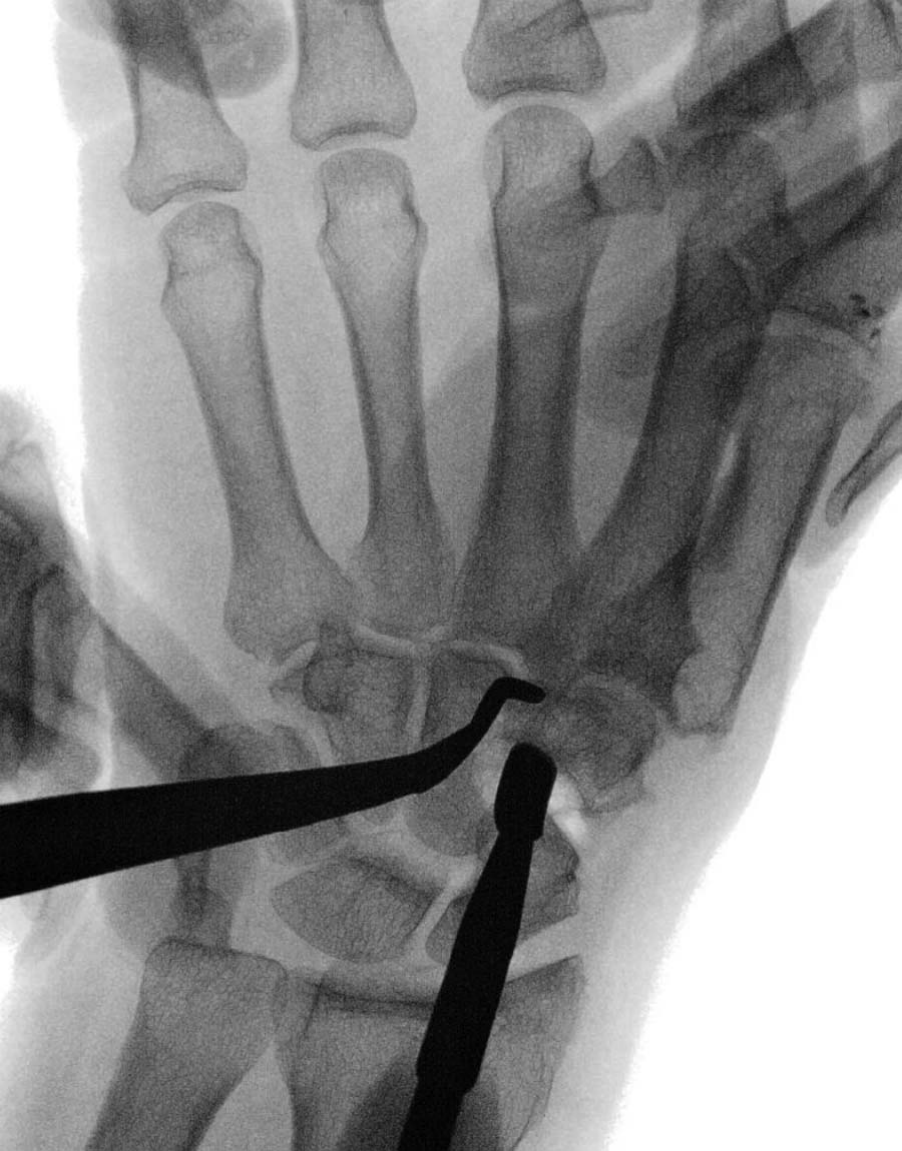
Intraoperative fluoroscopic view of the trapezium arthrolysis using periosteum elevator and 15 blade.

**FIGURE 7:**
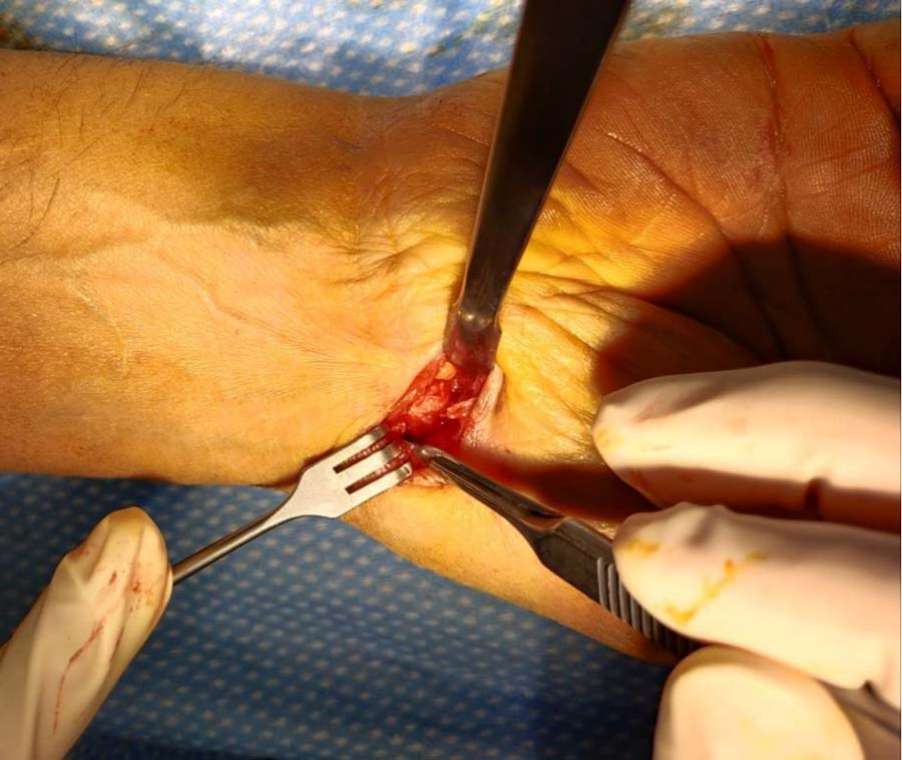
The trapezium arthrolysis.

Step 5: Proceed with trapezium arthrolysis using periosteum elevator and 15 blade (Figs. 6, 7).

**FIGURE 8:**
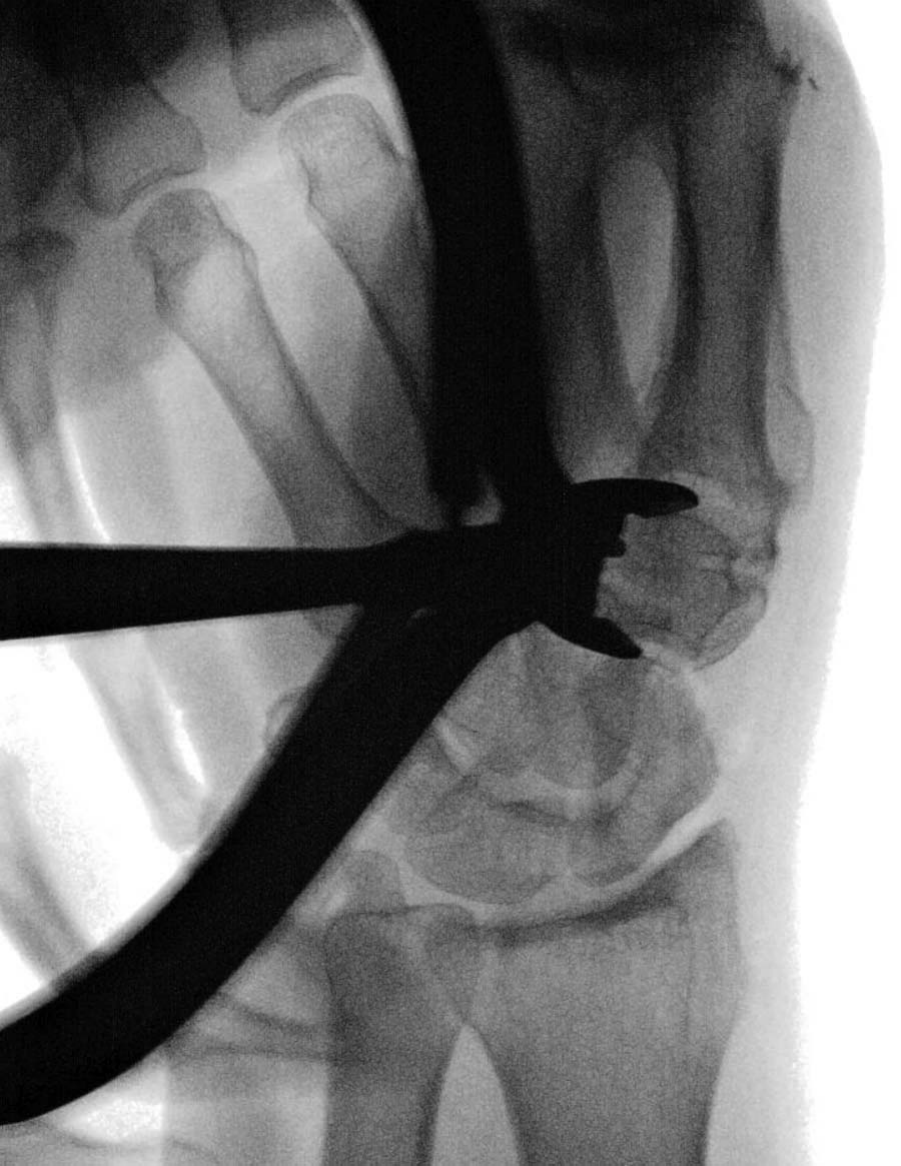
Intraoperative fluoroscopic view of the trapezium removing using a rongeur.

**FIGURE 9:**
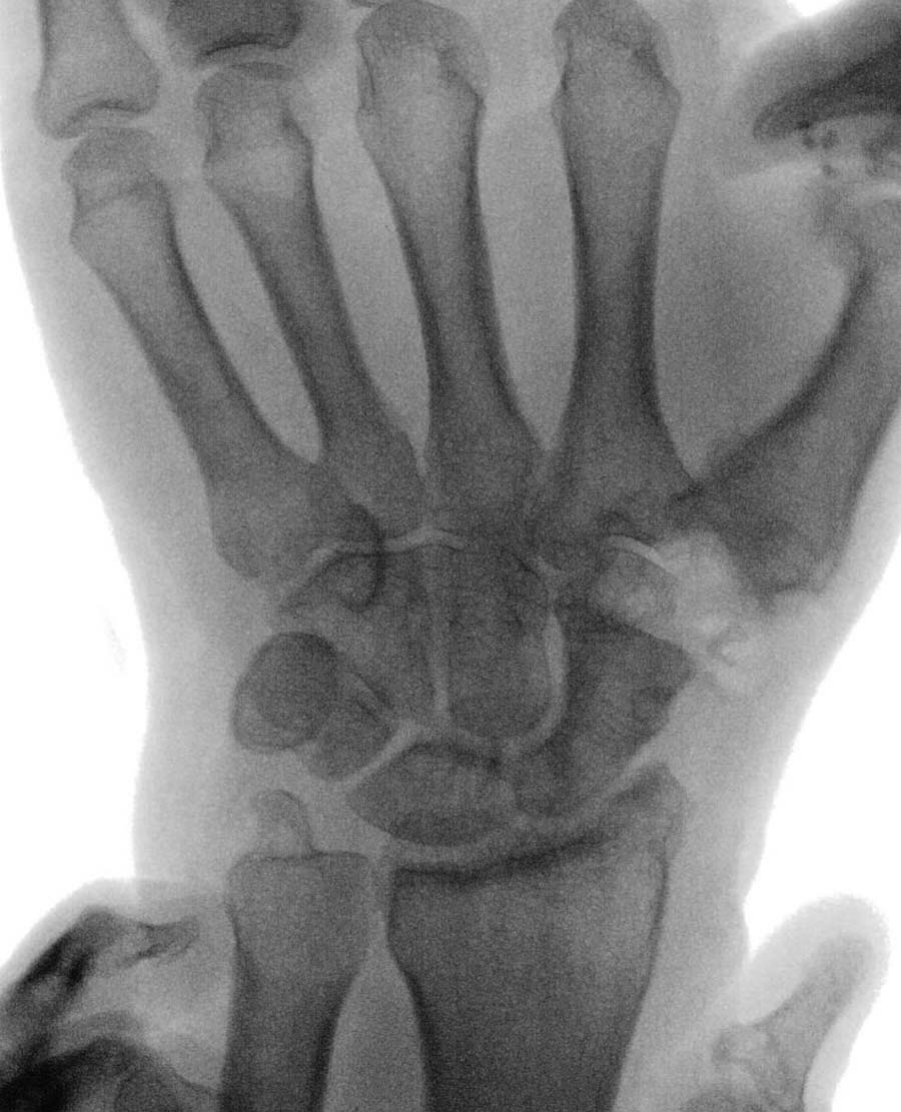
Intraoperative fluoroscopic view of the completed trapeziectomy.

Step 6: Remove the trapezium removed using a rongeur. The flexor carpi radialis and APL tendons were identified, and their insertion sites were carefully preserved as the entire trapezium was removed (Figs. 8, 9).

**FIGURE 10:**
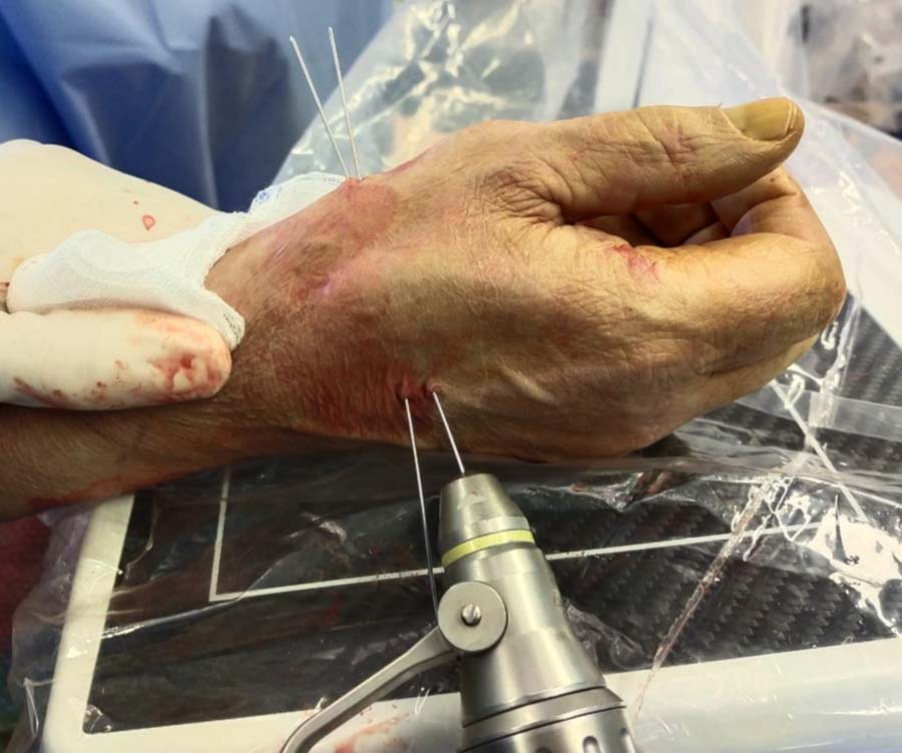
Insertion of two 1.0-mm diameter K-wires crosswise at the level of the surgical incision and along the first metacarpal ligament transition zone.

**FIGURE 11:**
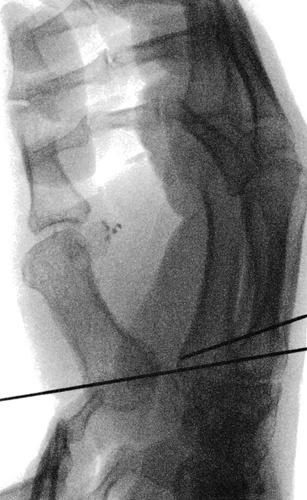
Positioning of two 1.0-mm diameter K-wires between the proximal dorsoulnar metaepiphyseal margin of the second metacarpal bone and the volar radial base of the first metacarpal bone, under fluoroscopic guidance.

Step 7: Under fluoroscopic guidance, two 1.0-mm diameter K-wires positioned between the proximal dorsoulnar metaepiphyseal margin of the second metacarpal bone and the volar radial base of the first metacarpal bone were inserted crosswise at the level of the surgical incision and along the first metacarpal ligament transition zone (Figs. 10, 11).

**FIGURE 12:**
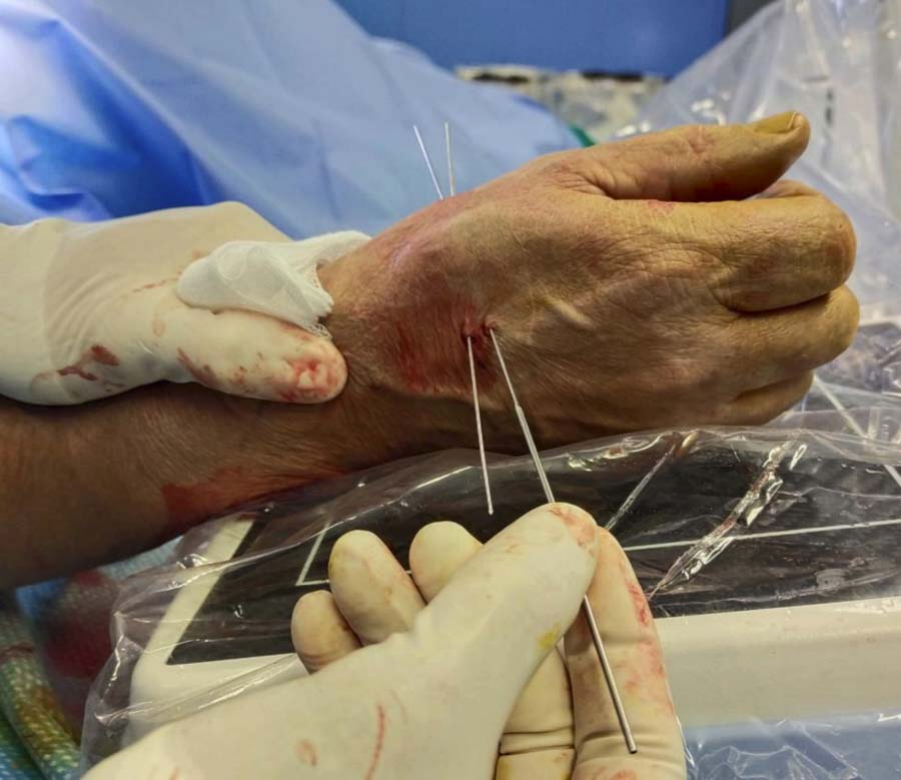
Inserting two 14-G needles (B-Braun Vasofix 14-G × 2″, 50 mm) using K-wires as a guide.

**FIGURE 13:**
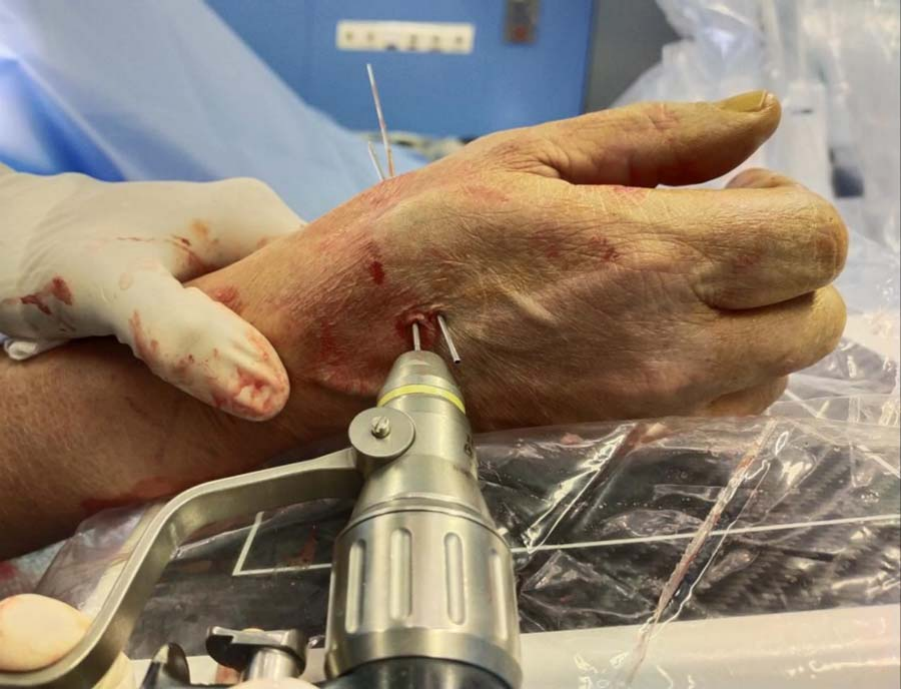
The 14-G needles insertion with hand drill, after a minidorsal incision performed along the previously inserted K-wire.

**FIGURE 14:**
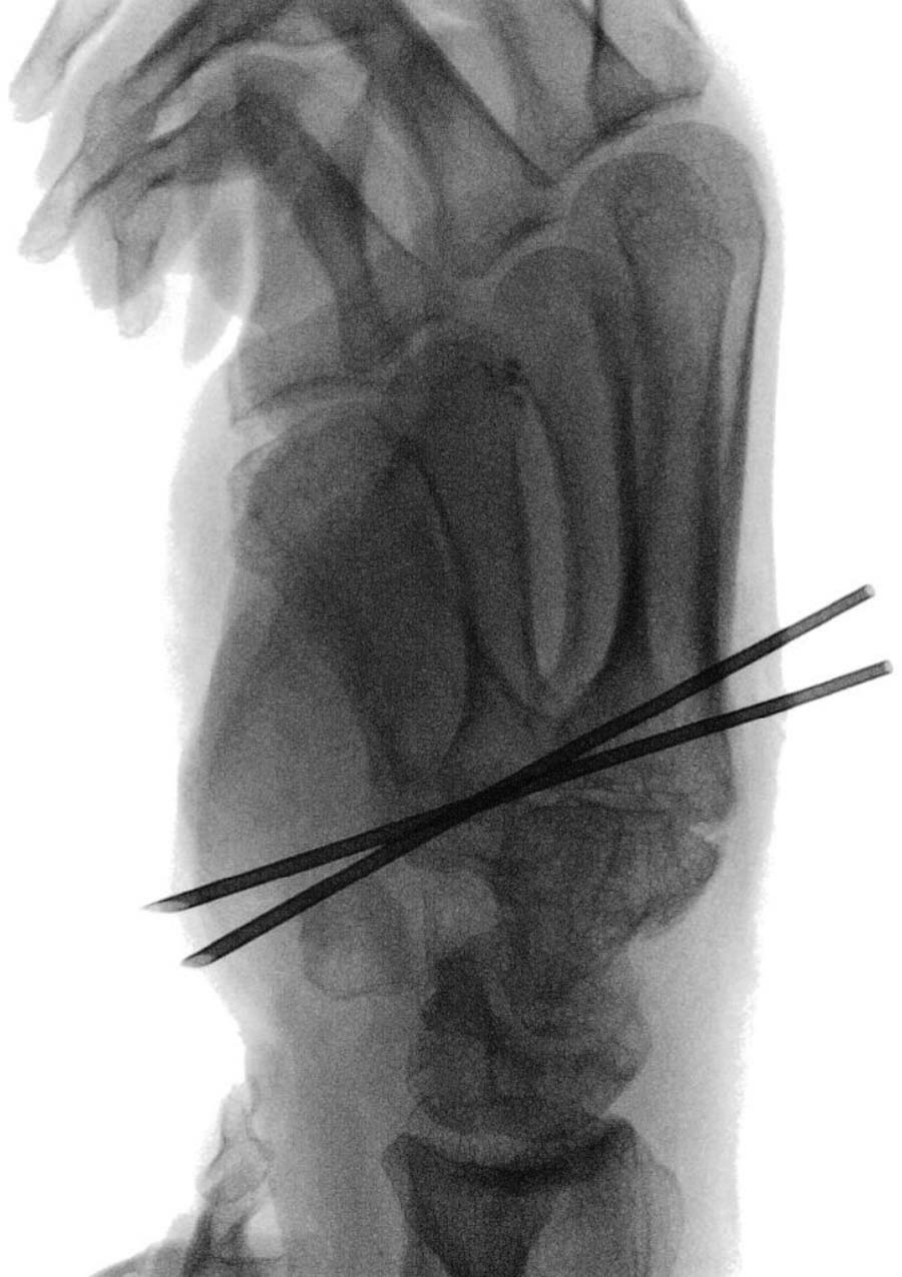
Intraoperative fluoroscopic view of the correct position of the 14-G needles.

Step 8: Using K-wires as a guide, two 14-G needles (B-Braun Vasofix 14-G × 2″, 50 mm) were inserted (Figs. 12, 13). Subsequently, a minidorsal incision of ~5 mm was performed along the previously inserted K-wire, protecting the superficial branches of the radial nerve. The 14-G needles can be inserted with a hand drill because the previously positioned K-wires keep the cannula empty from bone fragments. At the end of transosseous needle placement, the 2 K-wires were removed (Fig. 14).

**FIGURE 15:**
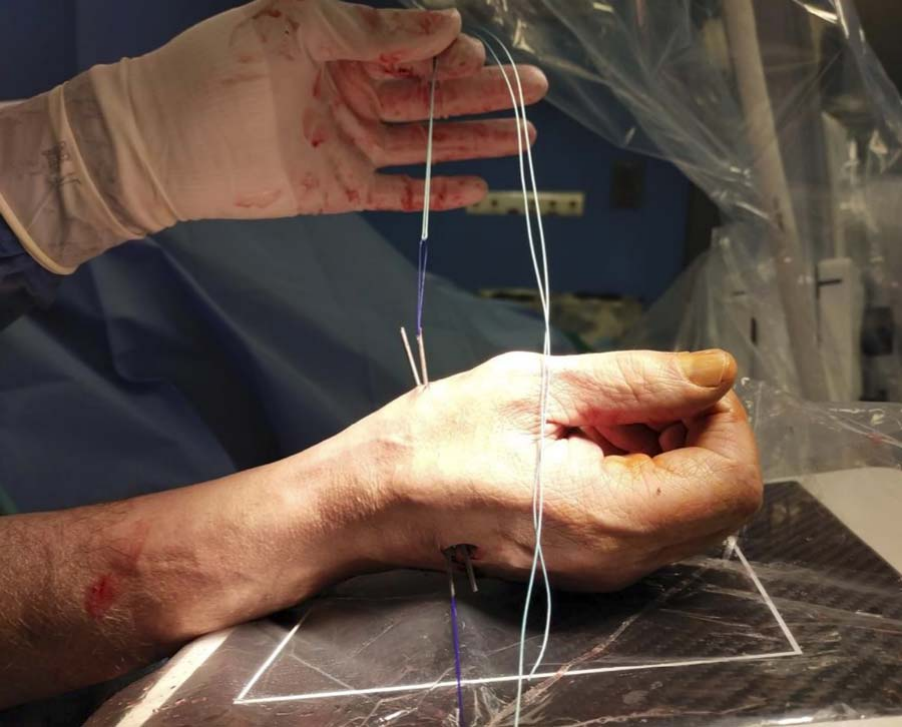
A loop made from Prolene 2/0.

**FIGURE 16:**
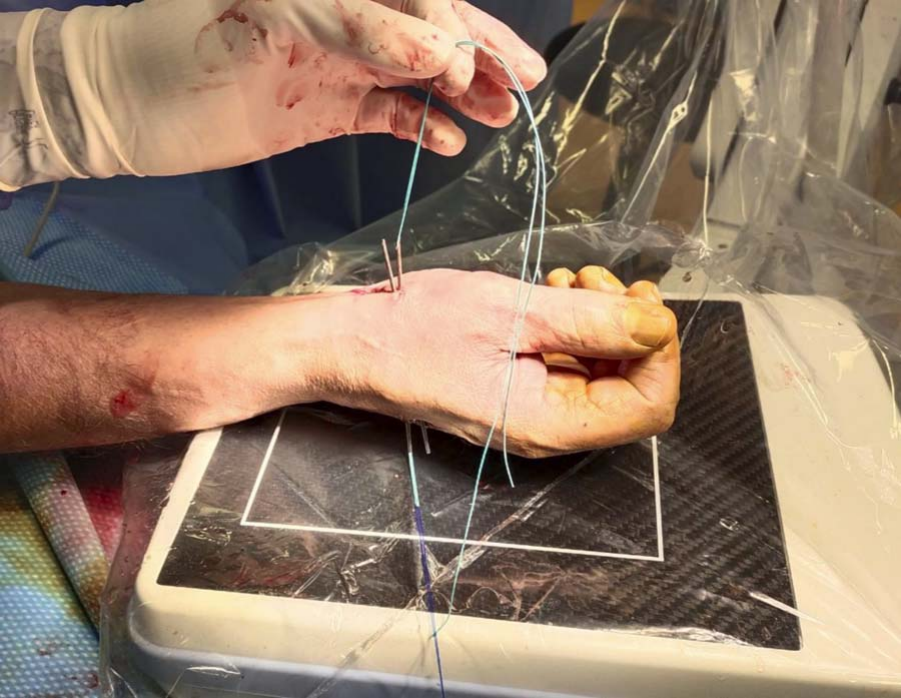
The Prolene 2/0 loop used to make a double-folded Ethibond #2 pass from volar to dorsal in the first 14-G needle.

**FIGURE 17:**
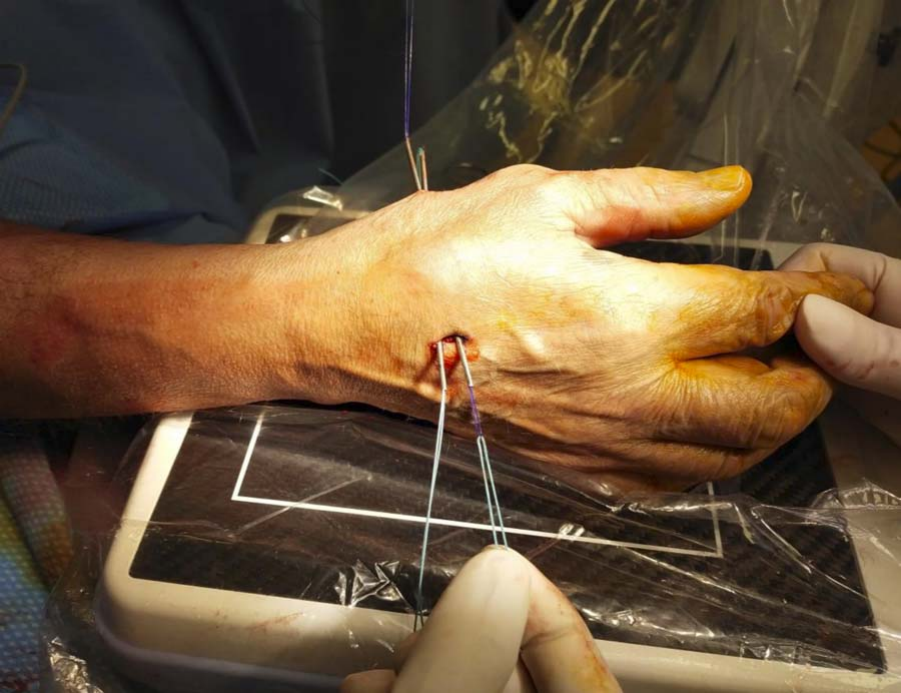
The same method used to pass the double Ethibond #2 in the second 14-G needle from dorsal to volar.

**FIGURE 18:**
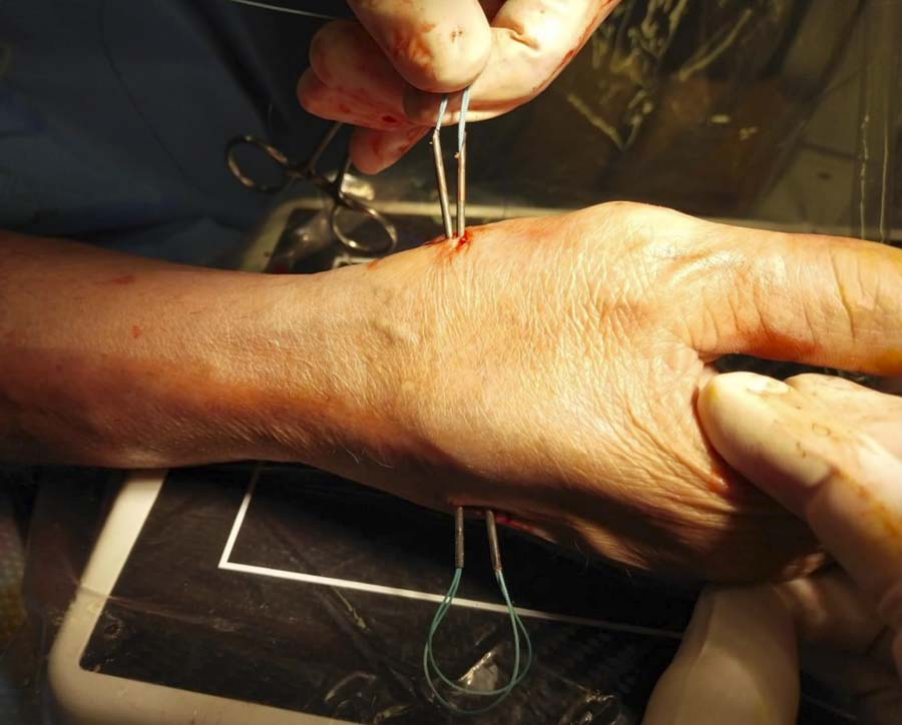
The 2 tales of the suture in the volar side.

Step 9: A loop was made from Prolene 2/0, which was used to make a double-folded Ethibond #2 pass, volar to distal, through the needle; the same method was used to pass the double Ethibond #2 from the second metacarpal to the first (Figs. 15–18).

**FIGURE 19:**
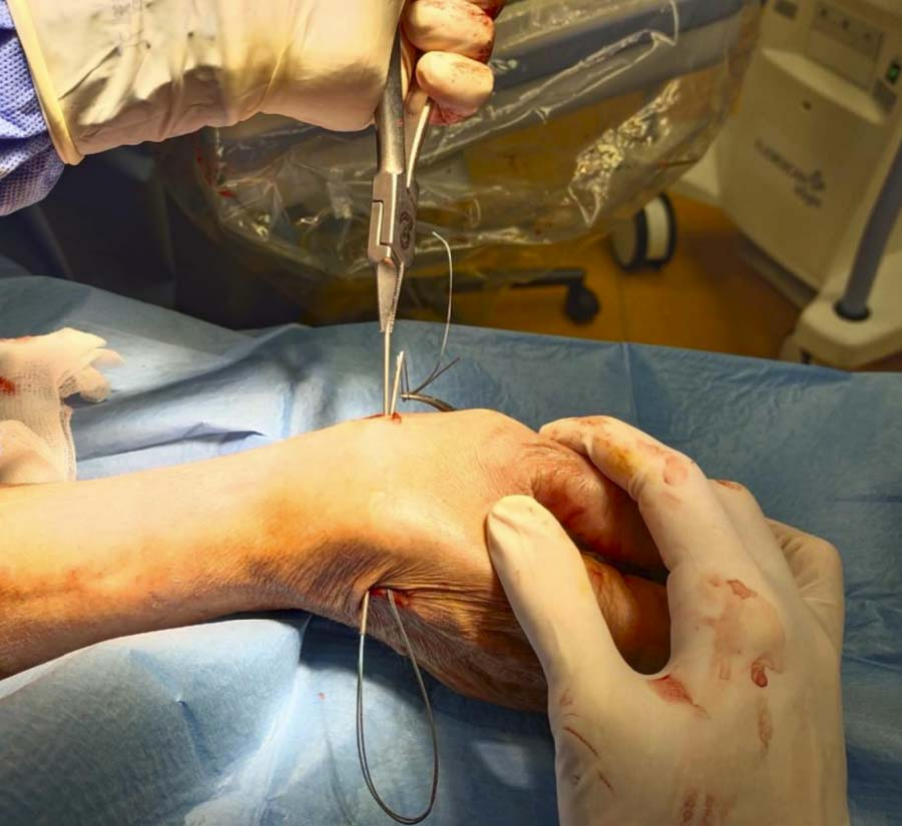
Removing of the 2 transosseous needles from the volar surgical access.

Step 10: The 2 transosseous needles were removed from the volar surgical access, taking care not to remove Ethibond #2 (Fig. 19). We recommend not using the drill to avoid the roll up of the Ethibond #2.

**FIGURE 20:**
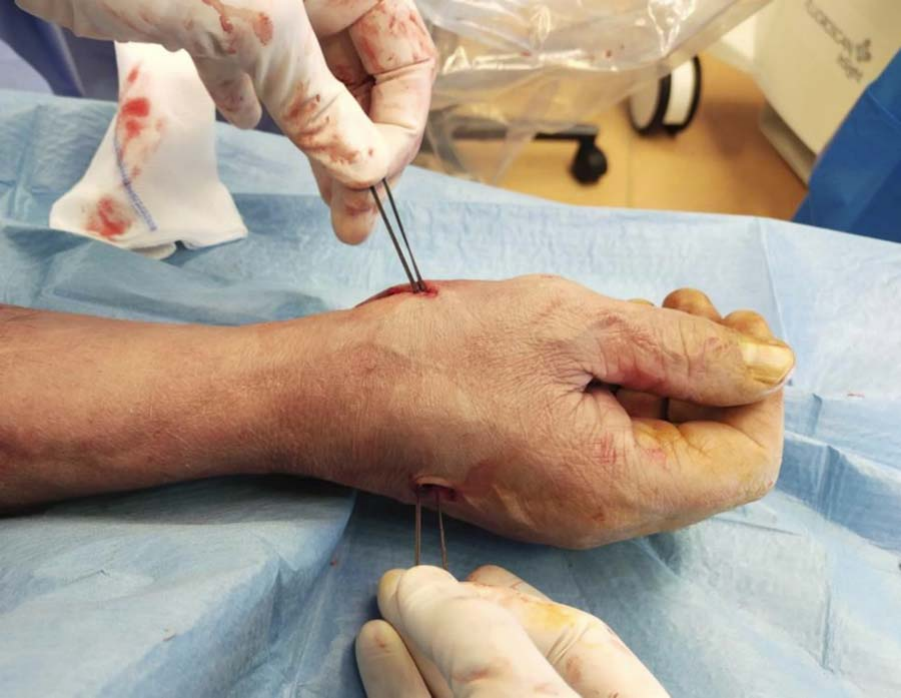
Position of the Ethibond #2 suture.

**FIGURE 21:**
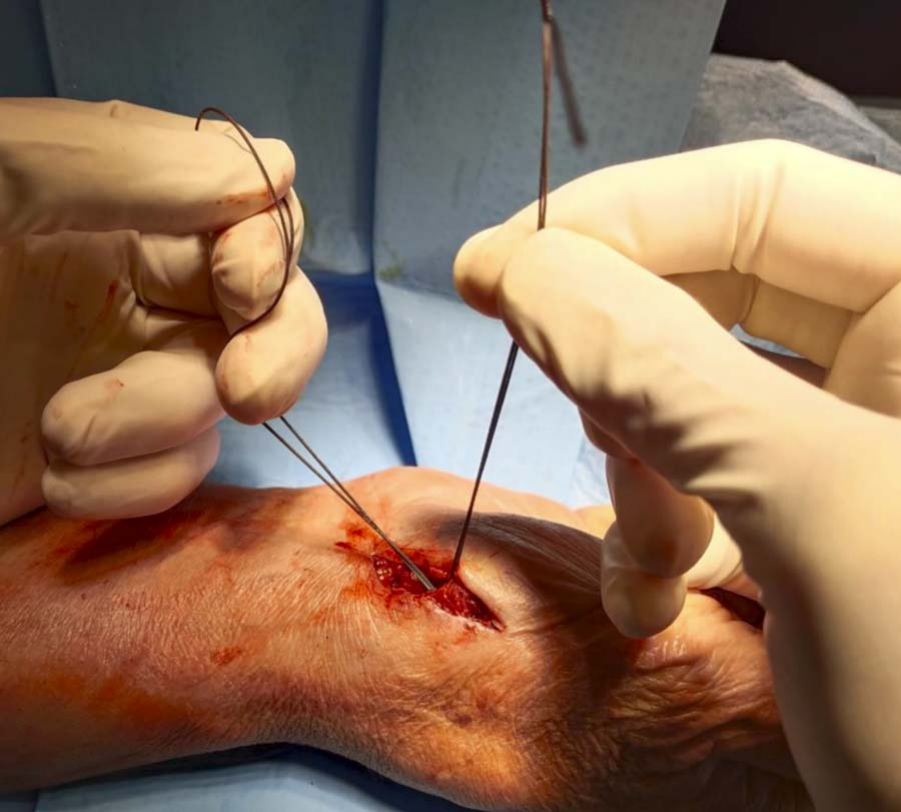
Suspension obtained applying a traction on the thumb and subsequently stabilized with a surgical knot between the first and second metacarpals.

**FIGURE 22:**
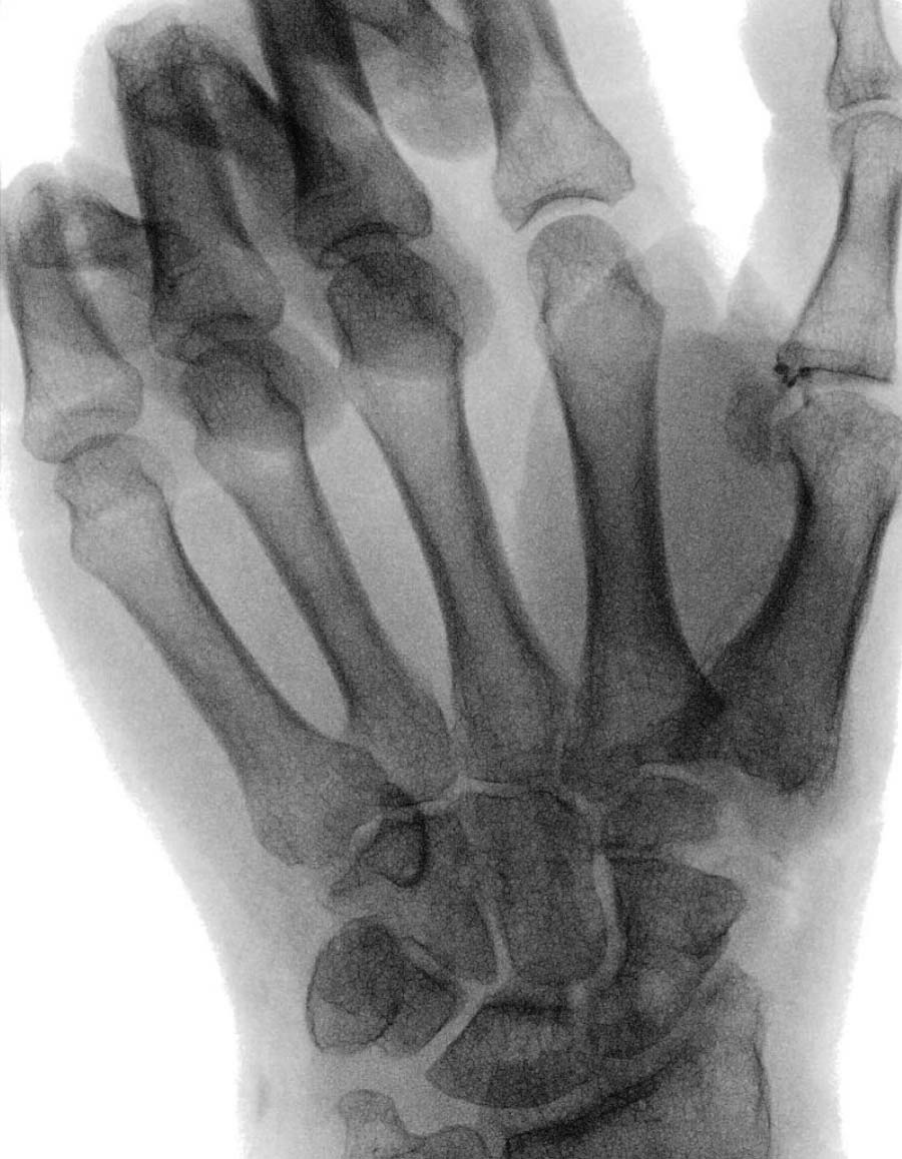
Intraoperative fluoroscopic view of the suspension.

Step 11: A suspension was made applying a traction on the thumb and subsequently stabilized with a surgical knot between the first and second metacarpals (Figs. 20–22).

Step 12: Hemostasis. Suturing of the joint capsule in Vycril 2/0. Resinsertion of the tenar musculature, at the distal tenar tendon tract of the thumb abductor muscle.

The patients were immobilized in a short-arm volar cast with thumb spica for 10 days. Thereafter, the cast was removed, a splint was worn, and gentle active and passive range of motion exercises were started.

## RESULTS

Between 2019 and 2020, the transosseous suture technique was performed on 14 patients (8 female and 6 male) with advanced OA of the TMC joint (Eaton-Littler stage II or III) at our institution. The right hand was involved in 7 patients and the left hand in 7. The mean age of patients was 60 years (range: 29–74).

The main finding of our study was that patients treated with the suture suspensionplasty technique had statistically significant improved outcome at 2-year follow-up, comparable to other peer reviewed techniques.. The mean outcomes are reported in Table 1. The average torniquet time is 45 minutes.

**TABLE 1:** Clinical Outcomes

	T0 preop	T1 3 mo	T2 6 mo	T3 1 y	T4 2 y
DASH score mean (SD)	67 (17)	30 (13)	24 (12)	22 (9)	21 (11)
VAS score mean (SD)	8 (1)	4.4 (1.6)	2.6 (1)	2 (1)	2 (1)
Kapandji test mean (SD)	7.5 (2)	8 (1)	9 (0.7)	9 (0.7)	9 (0.7)
Key Pinch mean (SD)	3 (1)	2.9 (1)	3.4 (1)	3.6 (1)	3.6 (1)

DASH indicates Disabilities of the Arm, Shoulder and Hand; VAS, Visual analogue scale.

## COMPLICATIONS

No complications were noted immediately after the procedure or during the 2-year follow-up period.

## DISCUSSION

Different comparative studies have failed to identify the best surgical technique for the treatment of TMC joint OA.^2,16,17^ In a randomized controlled study of 174 thumbs with a minimum of 5 years follow-up comparing hematoma and distraction arthroplasty, LRTI, and trapeziectomy with tendon interposition, no difference in outcomes was observed.^18^ Despite evidence, many hand surgeons still prefer LRTI over HAD,^19,20^ which could be associated with concerns regarding metacarpal subsidence after trapeziectomy alone.^4^ Among the suspension procedures available, SB suspensionplasty has become popular; this internal device allows thumb motion and rehabilitation to begin in the first 10 days; earlier mobilization leads to faster recovery^13^ and earlier return to daily activities and work. Biomechanical studies in cadaveric models have demonstrated that SB suspensions show a superior initial load-bearing profile and resistance to metacarpal subsidence over LRTI.^21,22^ Desai and colleagues compared the initial biomechanical strengths of SB suspensionplasty and LRTI in cadaveric models. They compared the average trapezial space height in both the SB suspensionplasty and LRTI groups during simulated physiological key pinch and incremental metacarpal load. The results of their study showed that SB suspensionplasty demonstrates greater resistance to metacarpal subsidence with immediate loading than LRTI.^22^ In their prospective cohort analysis, Shonuga et al^14^ found that short-term SB suspensionplasty had a significantly higher resistance to metacarpal subsidence than LRTI.

Otherwise, the practical concern of performing suspensionplasty with SB devices is its cost-effectiveness,^12,15^ particularly when compared with similar techniques using pins or tendon interpositions. Furthermore, SB also has some potential problems, such as fracture of the second metacarpal bone and irritation of soft tissue (tendon and dorsal radial nerve branches) due to the button. Overtensioning the system can be a source of pain due to impingement between the first and second metacarpal bases.^23^

Our study has some limitations. First, the sample size was small and the control group was not included. Future studies may overcome these limitations. Furthermore, a proper cost analysis was not performed; in our country, the SB has a cost of about 560 euros compared with the cost of 2 euros for each K-wire and 1.5 euros for Ethibond. Our technique has a longer average operative time compared with trapeziectomy and suspensionplasty with APL (45 vs. 35 min).

When we compared the results of our study to those of previous studies, we found similar objective and subjective outcomes (Table 2); we did not observe clinical complications described performing a SB, such as fracture of the second metacarpal or impingement between the first and second metacarpal. We believe that this was because of the lower rigidity of the construct.

**TABLE 2:** Clinical Results Compared With SB Clinical Results

Article	Patients	Mean follow-up (mo)	DASH QuickDASH	VAS	Kapandji	Key pinch (kg)	Complication	Revision
Our study	14	24	21	2.3	9	3.6	0	0
Yao et al^13^	14	64	9.2	—	9.6	107% of the controlateral side	3 (2 radial nerve neurapraxia 1 pain)	1
Yao et al^24^	21	33.6	10	—	—	86% of the controlateral side	2 (1 CRPS, 1 fracture of the second metacarpal)	—
Avant et al^12^	27	6	37	2	—	5	3 (1 infection, 2 palpable implant)	—
Dreant et al^25^	22 (8 revision surgery)	30	—	2	9	80% of the controlateral side	2 (1 infection, 1 device rupture)	2
Landes et al^15^	153 (arthroscopic)	14.5	—	—	—	95% of the controlateral side	1 osteomielitis	3
Ozcelik et al^26^	21 (arthroscopi)	50.1	5.5	1.9	9.2	20.2	1 loosening for direct trauma	1
Shonouga et al^14^	59	12	7.5	0.3	—	5.7	0	0
De George et al^27^	31	23.8	—	1.2	—	5.2	2 (1 CRPS, 1 palpable implant)	1

DASH indicates Disabilities of the Arm, Shoulder and Hand; CRPS, Complex Regional Pain Syndrome; VAS, Visual analogue scale.

Our techniques has some potential complications: irritation of the superficial branch of radial nerve on the second metacarpal, for this reason it is mandatory a blunt dissection in that area. Another potential complication is a breakage of the suture during the removal of the needles; in this case, the procedure need to be restarted from the K-wire positioning.

## CONCLUSIONS

Our surgical technique represents a viable alternative to SB, and is particularly attractive because of its cost-effectiveness.
